# No Evidence for Pathogenic Role of UBQLN2 Mutations in Sporadic Amyotrophic Lateral Sclerosis in the Mainland Chinese Population

**DOI:** 10.1371/journal.pone.0170943

**Published:** 2017-01-26

**Authors:** Xiao Huang, Shen Shen, Dongsheng Fan

**Affiliations:** Department of Neurology, Peking University Third Hospital, Beijing, China; Institute of Health Science, CHINA

## Abstract

Mutations in the *UBQLN2* gene, which encodes a member of the ubiquitin-like protein family (ubiquilin-2), have been identified in patients with dominant X-linked amyotrophic lateral sclerosis (ALS) and ALS with frontotemporal dementia (FTD). We analyzed mutations in the *UBQLN2* gene in a Chinese cohort of 515 patients with sporadic ALS (sALS). A novel missense mutation (p.M392V) was detected in one sALS patient. The p.M392V mutation substitutes a highly conserved residue, has not been reported in the population databases, and previously, at the same residue, a missense mutation p.M392I was detected in two Turkey ALS patients and was considered to be pathogenic, so the M392V is a variant of uncertain significance (VOUS) for ALS. We also found a deletion mutation (p.P500_G502del), which seems to be benign. In conclusion, our data suggest that mutations in the *UBQLN2* gene are rare in Chinese sALS patients.

## Introduction

It has been known that *UBQLN2* gene is associated with amyotrophic lateral sclerosis (ALS) and ALS with frontotemporal dementia (FTD) [1]. Studies of ALS cohorts from different ethnic groups have been conducted to identify the pathogenicity of *UBQLN2* mutations in ALS patients, and it is now recognized that *UBQLN2* mutations are not a common cause of ALS [2–7].

To date, Korea and Taiwan have conducted genetic surveys in ALS patients, and their results suggested that mutations in *UBQLN2* gene are rare [8, 9]. As for mainland China, a huge ethnic group of East Asian, few data are available. Thus, we assessed the site and frequency of *UBQLN2* gene mutations in a cohort of mainland Chinese patients with sALS.

## Methods

A total of 515 patients from mainland China were recruited in the study from January 2013 to December 2014. They were diagnosed with definite, probable or lab-supported probable ALS as per El Escorial criteria, and appeared to have sALS without clinical manifestation of FTD. Patients’ clinical data and blood samples were collected during their first visit to the outpatient department, and we have access to the information for identifying a special individual participant during the study. All patients had previously been screened for mutations in known ALS genes including SOD1, FUS, TARDBP, OPTN and DCTN1. All participants provided written informed consent in this study, which was approved by the Peking University Third Hospital Ethics Committee. Age- and sex-matched individuals without neurological disease were used as controls (n = 203).

The entire coding region of *UBQLN2* including the 5’ and 3’-UTR was amplified using five overlapping amplicons (S1 File) and further sequenced. Polymerase chain reactions (PCR) were performed using I-5TM 2X High-Fidelity Master Mix (CMLAB) as per manufacturer’s guidelines. PCR products were sequenced at Tsingke biological technology corporation. All sequence variations were confirmed by independent PCR reactions using primers from both forward and reverse directions. The PXX domain and the STI1 domain which contains the discovered missense mutation c.1174A>G were further sequenced in another 286 controls.

Discovered mutations were checked for presence in the public SNP databases dbSNP, the 1000 Genomes Project, Exome Variant Server (EVS) and Exome Aggregation Consortium (ExAC).

The potential impact of missense mutations on the structure and function of the encoded proteins was evaluated using two bioinformatic prediction programs: PolyPhen-2 and SIFT. To assess whether the altered amino acids were conserved, orthologous peptide sequences were obtained for the human UBQLN2 gene from five different species and aligned using the CLUSTALW2 server.

## Results

**Fig 1:**
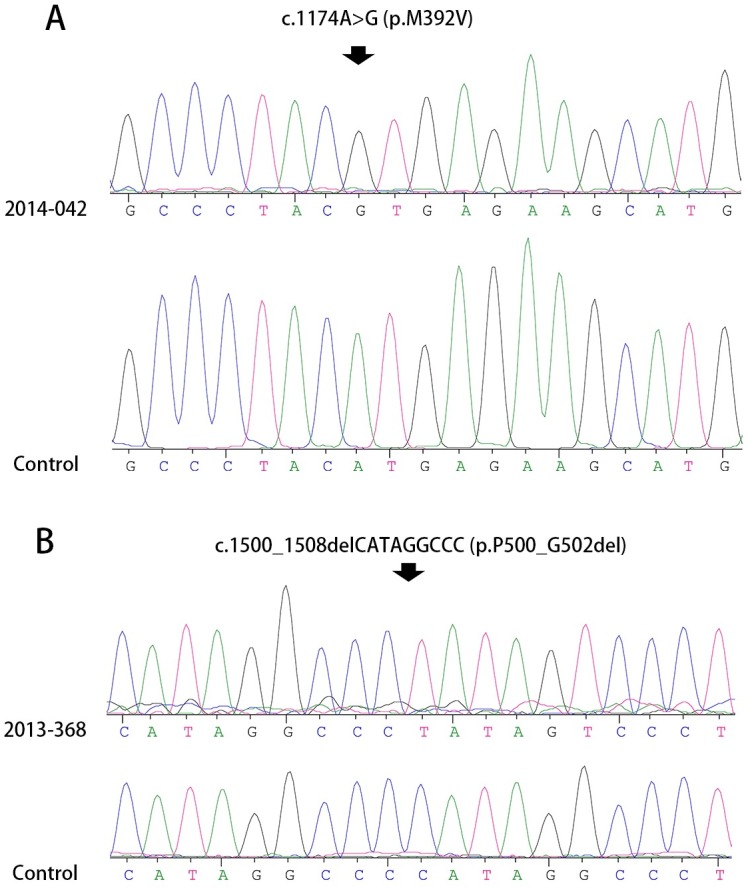
UBQLN2 mutations in Chinese sALS patients. (A) Patient 2014–042 had a c.1174A>G (p.M392V) missense mutation. (B) Patient 2013–368 carried a deletion mutation (c.1500_1508delCATAGGCCC, p.P500_G502del).

We found a missense mutation (c.1174A>G; p.M392V) of *UBQLN2* in one sALS patient (2014–042) and a deletion mutation (c.1500-1508delCATAGGCCC; p.P500_G502del) in a patient with sALS (2013–368) (Fig 1). None of these two variants were found in the 489 (203+286) healthy controls, and the two residues were both in conserved domains. As for clinical information, patient 2014–042 was a 62-year-old man who presented with a 32-month history of progressive upper limb weakness. The patient was diagnosed as ALS-FAS and had an amyotrophic lateral sclerosis function rating scale-revised (ALSFRS-R) score of 46 at diagnosis. In a two years follow-up visit, the patient now has an ALSFRS-R score of 35 and shows no sign of FTLD symptoms. Patient 2013–368 was a 63-year-old man with a 20-month history of slowly progressive limb weakness and had an ALSFRS-R score of 47 at diagnosis. At the time of report, the patient has an ALSFRS-R score of 44 and showed no sign of FTLD symptoms. In addition, we also detected a silent variant in one patient and one control: c.51T>A (p.P17P) (2/718; rs371163085), which seems to be a benign polymorphism.

## Discussion

**Fig 2:**
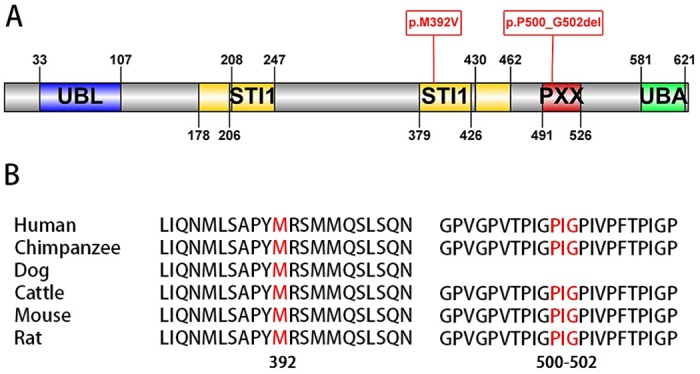
The location and genetic conservation of the mutations in *UBQLN2* gene. (A) Ubiquilin-2 protein indicating structural and functional domains: UBL (ubiquitin-like domain), STI1 (heat-shock-chaperonin binding motif), PXX (proline-rich region) and UBA (ubiquitin-associated domain). Our detected mutations are displayed in red. (B) The conservation of ubiquilin-2 protein in different species. The mutated residues are displayed in red.

The missense variant we detected, p.M392V, is a novel variant of uncertain significance (VOUS), as it was predicted to be possibly damaging (by Polyphen-2) or tolerated (by SIFT). The patient’s mother died at the age of 60 while his father was still alive at the time of report, and both of them did not show signs of weakness or atrophy. It is a pity that we did not get the DNA samples of the parent’s parents to investigate the transmission of the mutation. Interestingly, in the same position, a mutation p.M392I was detected in Turkey ALS patients, and it was thought to be deleterious [7]. However, the onset age of the patients carrying the p.M392I mutation were fairly young (14 and 16 years old) and one of them showed a special subtype of ALS: Madras-type MND. By contrast, our patient showed a typical ALS-FAS phenotype. This may be due to that different amino acid change could lead to a different phenotype. None of these two mutations were found in public SNP resources and in controls. So, according to the ACMG Standards and guidelines for the interpretation of sequence variants [10], the p.M392V variant is a VOUS for ALS. The mutation is located at a quite conservative region (Fig 2), so further analyses are needed to clarify the pathogenicity of the p.M392V variant of UBQLN2, for example, to test the parental samples for the variant or functional studies of the p.M392V protein.

The deletion mutation, c.1500_1508.delCATAGGCCC, was firstly reported by Millecamps in a French fALS female proband. The proband’s mutation was at the heterozygote state, and her father, also an ALS patient, did not carry this mutation. Rather, they detected the mutation at the heterozygous state in a female control subject, so the mutation did not seem to be deleterious [3]. Besides, just before the mutation side, the p.P497_G499del mutation, which causes same amino acids change, was mapped in the ExAC database (global 10/55335, East Asian 2/4156). This further confirmed that the mutation seems to be benign for ALS.

In conclusion, the present study demonstrates the low frequency of *UBQLN2* variants in mainland Chinese sALS patients. Previously, it has been estimated that *UBQLN2* gene accounts for less than one percent of sALS in populations of European ancestry [11]. As for the Asian region, Kim et al. reported a missense variant (c.942T>A, p.D314E) existing both in 2 unrelated sALS patients and 5 healthy control individuals of Korean population, indicating that this variant might be a rare polymorphism [8]; and no Taiwanese patient was found to have any mutation of *UBQLN2* gene in Soong’s research [9]. Thus, our conclusion is in accordance with the results of globe and the Asian region, suggesting that causative mutations are rare in the *UBQLN2* gene in sALS patients. We did not find an explicit genotype-phenotype association in sALS patients with *UBQLN2* mutations, but we assume that different mutation locus and different amino acid change of *UBQLN2* gene can lead to diverse clinical manifestations. Further functional study is need to elucidate the pathogenicity of the p.M392V missense variant of *UBQLN2* gene.

## Supporting Information

S1 File The sequence of primers used in the study (from Deng et al., 2011). The amplified five overlapping PCR fragments cover the entire coding sequence (1,872bp), 125bp of the 5’-UTR and 293bp of the 3’-UTR.
(DOC)
