# Supplementary material for: No Evidence for Pathogenic Role of UBQLN2 Mutations in Sporadic Amyotrophic Lateral Sclerosis in the Mainland Chinese Population
Source: PLoS One. 2017 Jan 26;12(1):e0170943. doi: 10.1371/journal.pone.0170943 (PMC5268382; doi:10.1371/journal.pone.0170943)
Supplement: S1 File — The amplified five overlapping PCR fragments cover the entire coding sequence (1,872bp), 125bp of the 5’-UTR and 293bp of the 3’-UTR. (DOC) [file pone.0170943.s001.doc]

## Supplemental Text

## The sequence of primers used in the study (from Deng et al., 2011).

The amplified five overlapping PCR fragments cover the entire coding sequence (1,872bp), 125bp of the 5’-UTR and 293bp of the 3’-UTR.

UBQLN2-1F: 5′-cttcatcacagaggtaccgtg-3′; UBQLN2-1R: 5′-gtgtggagttactcctgggag-3′

UBQLN2-2F: 5′-catgatgggctgactgttcac-3′; UBQLN2-2R: 5′-ctcttgtgcggcattcagcatc-3′

UBQLN2-3F: 5′-gacctggctcttagcaatctag-3′; UBQLN2-3R: 5′-gtgtctggattctgcatctgc-3′

UBQLN2-4F: 5′-cacagatgatgctgaatagcc-3′; UBQLN2-4R: 5′-gctgaatgaactgctggttgg-3′

UBQLN2-5F: 5′-ctgcacctagtgaaaccacgag-3′; UBQLN2-5R: 5′-aacagcattgattcccaccac-3′
